# A Methodological Approach to Assessing the Health Impact of Environmental Chemical Mixtures: PCBs and Hypertension in the National Health and Nutrition Examination Survey

**DOI:** 10.3390/ijerph8114220

**Published:** 2011-11-09

**Authors:** Krista L. Yorita Christensen, Paul White

**Affiliations:** US Environmental Protection Agency, Mailstop 8623P, 1200 Pennsylvania Ave NW, Washington, DC 20460, USA; E-Mail: white.paul@epa.gov

**Keywords:** cumulative risk assessment, PCBs, hypertension

## Abstract

We describe an approach to examine the association between exposure to chemical mixtures and a health outcome, using as our case study polychlorinated biphenyls (PCBs) and hypertension. The association between serum PCB and hypertension among participants in the 1999–2004 National Health and Nutrition Examination Survey was examined. First, unconditional multivariate logistic regression was used to estimate odds ratios and associated 95% confidence intervals. Next, correlation and multicollinearity among PCB congeners was evaluated, and clustering analyses performed to determine groups of related congeners. Finally, a weighted sum was constructed to represent the relative importance of each congener in relation to hypertension risk. PCB serum concentrations varied by demographic characteristics, and were on average higher among those with hypertension. Logistic regression results showed mixed findings by congener and class. Further analyses identified groupings of correlated PCBs. Using a weighted sum approach to equalize different ranges and potencies, PCBs 66, 101, 118, 128 and 187 were significantly associated with increased risk of hypertension. Epidemiologic data were used to demonstrate an approach to evaluating the association between a complex environmental exposure and health outcome. The complexity of analyzing a large number of related exposures, where each may have different potency and range, are addressed in the context of the association between hypertension risk and exposure to PCBs.

## 1. Introduction

Humans are exposed to a multitude of environmental exposures, and these exposures may interact with one another to alter risk for health outcomes. In 2008, the National Research Council issued a report urging risk assessors to consider chemical mixtures rather than each component in isolation, when considering effects on human health [1]. However, there is currently no agreed upon strategy for taking into account multiple chemicals in epidemiologic studies. One issue is the dimensionality of the data involved—sample size may be limited, but numerous covariates, including multiple chemical exposures, must be investigated. Further, the varying ranges in the values of the exposures being assessed, and non-independence of the exposures make it difficult to determine relative contributions of each exposure component.

These issues are relevant when assessing human health effects of exposure to polychlorinated biphenyls (PCBs). PCBs are a class of commercially synthesized chemicals, and consist of 209 different congeners. Although PCBs have a wide range of industrial applications, the most prevalent use is in electronics manufacture. Production of PCBs was banned in the 1970s due to concerns about their toxic effects, but because of their persistence, many PCBs are still detectable in the US population. The main source of exposure for the general population is diet; PCBs bioaccumulate up the food chain, concentrating in some fish, meat, and poultry products. PCB exposure in humans has been linked to numerous adverse health outcomes, including increased risk of elevated blood pressure, or hypertension. However, as noted in a recent review article, findings have been mixed in both cross-sectional and longitudinal studies [2]. For example, in a 24-year follow up of the Yucheng cohort, Wang *et al.* reported a non-significant 20–40% increase in hypertension risk among exposed persons; results were not presented stratified by congener (or congener group) or level of exposure [3]. In contrast, Goncharov *et al.* report significant associations between hypertension and serum PCBs, as well as a positive association between PCBs and blood pressure even in the normotensive range, in a cross-sectional study of persons living near a PCB production facility [4]. The association varied depending on congener grouping—significant associations were noted for total PCBs, di-*ortho*, tri-*ortho* and tetra-*ortho* PCBs, but not for estrogen-like and mono-*ortho* PCBs. These examples emphasize the importance of evaluating the effects of PCBs based on mechanism and/or structure with respect to health outcomes.

In this work we used data from a nationally representative survey to describe one approach to such an epidemiologic analysis, using as our case study the association between hypertension and serum PCBs.

## 2. Experimental Section

Data from the 1999–2000, 2001–2002, and 2003–2004 cycles of the National Health and Nutrition Examination Survey (NHANES) [5] were obtained from the publicly available website maintained by the National Center for Health Statistics [6]. The NHANES is a nationally representative, complex sample survey of the civilian, non-institutionalized US population. For this analysis, we included all participants aged ≥20 years. Serum concentrations of PCBs were assessed in a randomly selected 1/3 subsample of NHANES participants. Multiple PCBs were measured in each NHANES cycle, and 22 different congeners were measured across all 3 cycles: PCB 52, 66, 74, 99, 101, 105, 118, 128, 138&158 [co-eluting], 146, 153, 156, 157, 167, 170, 172, 177, 178, 180, 183 and 187. Serum samples were analyzed using mass spectrometry, and both whole-weight and lipid-adjusted values are reported; for these analyses, we used whole weight values, and included lipid concentration (estimated as 2.27*total cholesterol + triglycerides + 62.31) as a covariate in our analyses. In the NHANES dataset, serum concentration values below the limit of detection (LOD) are replaced by (LOD/√2).

Hypertension was defined as meeting any of the following three criteria: answering ‘yes’ to the question “Have you ever been told by a doctor or other health professional that you had hypertension, also called high blood pressure?”; answering ‘yes’ to the question “Because of your (high blood pressure/hypertension), have you ever been told to take prescribed medicine?” or reporting taking a prescription medication classified as an antihypertensive; measured systolic pressure ≥140 mm Hg or measured diastolic pressure ≥90 mm Hg (average of three readings).

Based on the literature on risk factors for hypertension, the following variables were considered to be potential confounders: age at interview, sex, race/ethnicity, BMI, smoking, physical activity, family history of CVD, total cholesterol, and serum lipid concentration. Information on age, sex, and race/ethnicity was also collected during the household interview. Race/ethnicity was reported as one of 5 categories: non-Hispanic White, non-Hispanic Black, Mexican American, other Hispanic, or Other (which includes mixed and unknown race). Body mass index (BMI) was calculated from height and weight measured during the medical exam. Participants were classified as either a current smoker or not a current smoker, and as undertaking regular physical activity (either moderate or vigorous physical activity in the past 30 days) or not. Finally, participants were considered to have a family history of cardiovascular disease if either parent experienced a myocardial infarction before the age of 50.

All analyses were performed in SAS v.9.1. As our goal is to illustrate a methodological approach rather than test a hypothesis regarding the true association between PCB exposure and hypertension in the general population, we did not account for the complex survey design of the NHANES in these analyses—results are unweighted and do not include the survey design variables. The association between PCB serum concentrations and hypertension was first assessed using ‘traditional’ epidemiologic methods, in an unconditional multivariate logistic regression model, controlling for the potential confounders described. Serum lipids were included as a separate covariate rather than using lipid-adjusted PCB concentrations, because serum lipids may be related to the outcome of hypertension; in this scenario, using lipid-adjusted concentrations may introduce additional bias [7,8]. However, we did also perform sensitivity analyses using lipid-adjusted values for comparison.

PCBs were considered individually (one congener at a time), as well as in groups. The groups are defined as in Goncharov *et al*. and include estrogenic PCBs (PCBs 66, 74, 99, 128), mono-*ortho* substituted PCBs (66, 74, 105, 118, 156, 157, 167), di-*ortho* substituted PCBs (52, 99, 101, 128, 138, 146, 153, 170, 172, 180), and tri- or tetra-*ortho* substituted PCBs (177, 178, 183, 187) [4]. In addition, dioxin-like PCBs (105, 118, 156, 157, 167, 170, 180) were also assessed as a group.

The association between odds of hypertension and PCB exposure was assessed by modeling serum concentration of PCBs both as categorical and as continuous exposures. In the categorical analysis, exposure was categorized according to either quartiles (grouped congeners) or as below the LOD (referent), compared to tertiles of the values above the LOD (individual congeners). When treating serum concentration as a continuous variable, results are shown for the untransformed exposure variable, as well as after natural log transformation and after standardization ([value – sample mean]/sample standard deviation). Natural log transformation is commonly used to improve normality of skewed variables, while standardization allows the comparison of effect estimates across exposures which have different ranges. Potential non-linearity of the exposure-response curve was investigated using generalized additive models (GAMs). GAMS are used to fit smooth non-parametric functions to continuous predictor data. The GAM procedure in SAS separates the linear and non-linear components of the relationship between the continuous exposure (PCB serum level) and binary outcome (hypertension), adjusting for multiple covariates.

Next, we investigated the joint association of PCBs with hypertension, including all PCB congeners in the regression model (along with potential confounders). Three considerations are noted for this model. First, the large number of predictors relative to the sample size (and number of cases) could lead to quasi- or complete separation of points and numerical instability. Second, the congeners vary in scale due to different ranges of exposure and accumulation. Third, congeners may be correlated with each other, which may lead to difficulty disentangling independent effects. Therefore, we sought to assess correlation/multicollinearity, and identify those congeners which were most informative with respect to risk of hypertension. First, we constructed a model including all the PCB congeners as predictors and hypertension status as an outcome, and used the collinearity diagnostics in the REG procedure to determine groups of highly correlated congeners. Cluster analysis (PROC VARCLUS) and discriminant analysis (PROC STEPDISC) were used to identify clusters of ‘similar’ PCBs, and most informative PCBs respectively. Principal component analysis (PROC PRINCOMP) was used to construct new predictor variables from linear combinations of the PCB concentrations. Finally, we used an optimization approach to identify the most informative PCB congeners, similar to that described by Gennings *et al.* [9]. For this method, all standardized PCBs were included in a logistic regression model along with potential confounders. The NLP (non-linear programming) procedure was used to construct a weighted sum of the centered congeners which maximized the log-likelihood of the logistic regression model. Weights were constrained to take values between 0 and 1, with the weights summing to one. Under these constraints, the weight assigned to a given congener is representative of the strength of the association between that congener and risk of hypertension, controlling for covariates and the other PCBs.

## 3. Results and Discussion

### 3.1. Results

Characteristics of the study population are given in Table 1. Out of 31,126 persons screened for the 1999–2004 NHANES cohorts, 49.3% (n = 15,332) were aged 20 or older. Of these, 29.3% (n = 4,496) were included in the laboratory assessment of PCBs. However, 377 had missing values for all PCB analytes, and were therefore not included in further analyses, leading to a final sample of 4,119 individuals. In this population, 43.9% met one of the three criteria to be categorized as hypertensive. Among these, 72.3% reported having had a physician diagnosis of hypertension, 67.6% reported taking an antihypertensive medication, and 52.9% had elevated blood pressure during the medical exam. Nearly one-fifth of those classified as hypertensive had only elevated blood pressure (no diagnosis or antihypertensive medication; 19.7%). The systolic and diastolic blood pressure readings for these individuals were generally borderline, with means of 152.4 (SD = 78.6, interquartile range [IQR]: 142.0–161.0) and 78.6 (SD = 17.6, IQR: 70.0–90.7), respectively. Among those with a physician diagnosis of hypertension, 82.5% were on antihypertensive medication. On average, those with hypertension were older (mean age of 60.8 [SD = 16.7] years compared to 40.4 [SD = 15.6] years) and had higher BMIs (mean BMI of 29.5 [SD = 6.4] compared to 27.1 [SD = 5.7]). The average total cholesterol among those with hypertension was somewhat higher compared to normotensive individuals (206.3 [SD = 44.2] mg/dL compared to 198.3 [SD = 41.9] mg/dL), as were the average systolic and diastolic blood pressures (141.3 [SD 22.8] mm Hg compared to 114.6 [SD = 11.3] mm Hg, and 73.1 [SD = 17.0] mm Hg compared to 68.5 [SD = 10.8] mm Hg, respectively).

In multivariate logistic regression analysis, the covariates most strongly associated with risk of hypertension were age, race/ethnicity and BMI (all p-values < 0.0001); strong associations were also noted for serum lipids (p-value = 0.0015) and family history of CVD (p-value = 0.0021). Compared with non-Hispanic whites, non-Hispanic Black race/ethnicity was associated with higher risk (OR = 2.04, 95% CI: 1.64–2.54), while Mexican-American race/ethnicity was associated with reduced risk (OR = 0.78, 95% CI: 0.64–0.96). A 10-year increase in age was associated with an OR of 2.09 (95% CI: 1.98–2.20). Physical activity (p-value = 0.14), total cholesterol (p-value = 0.11), current smoking (p-value = 0.86) and sex (p-value = 0.60) were not strongly associated with risk of hypertension. Regardless, all of these covariates were retained in further analyses as a priori selected potential confounders.

The highest PCB concentrations were seen for PCBs 153, 180 and 138 (geometric means [GMs] of 0.20, 0.15, and 0.14 ng/g, respectively; Table 2). Serum concentration of total PCBs were similar by gender (GMs of 1.1 ng/g for both males and females), increased with age (GM of 0.6 in those aged 20–39 compared to 2.3 in those aged 70 or older), and were highest among non-Hispanic Black and non-Hispanic White participants (GMs of 1.3 and 1.2 ng/g, respectively). Total PCBs also varied by body mass, with the lowest levels seen among those in the normal weight category (18.5 ≤ BMI < 25: GM of 1.0 ng/g) and the highest among those in the overweight (25 ≤ BMI < 30) and underweight (BMI < 18.5) categories (GM of 1.2 for both); those in the obese category had a GM of 1.1 ng/g. Finally, participants with hypertension had higher total PCB levels compared to non-hypertensive subjects (GMs of 0.8 and 1.6 ng/g, respectively).

**Figure 1:**
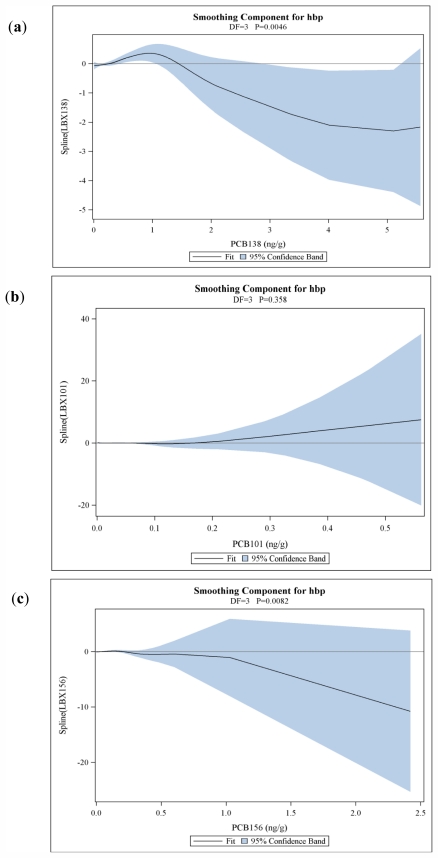
Partial prediction plots for PCBs (**a**) 101, (**b**) 138, and (**c**) 156, using GAM models adjusted for age in years, sex, BMI at exam, race/ethnicity, current smoking status, regular physical activity status, family history of cardiovascular disease, total cholesterol and serum lipid concentration.

Table 3 presents the results of multivariate logistic regression analysis, where each PCB or congener grouping is included one at a time in a model with potential confounders. There was an increased odds of hypertension among those in the highest compared to the lowest quartile of total PCBs (OR = 1.38, 95% confidence interval [CI]: 1.02–1.87). The association was of borderline significance when treating total PCB serum concentration as a continuous variable (beta = 0.0689 [SE = 0.0360]). Congener groups based on activity (estrogenic, dioxin-like) and structure (mono-*ortho*, di-*ortho*, tri- and tetra-*ortho* substituted) were also significantly associated with increased risk of hypertension, although results varied with treatment of the exposure variable (continuous or categorical). In the congener-specific models, there was a significant association between risk of hypertension and numerous PCBs, although in some cases the effect estimates from categorical and continuous models showed opposing effects—a decreased risk of hypertension associated with the middle quartiles of exposure but increased risk predicted from the continuous exposure model. For example, PCB 66 was significantly associated with increased odds of hypertension when treated as a continuous variable; however, odds ratios for the increasing tertiles of exposure above the LOD were 0.95, 0.77, and 1.13. It is possible that the significant association in the linear exposure model is driven by the change from the second to the third tertile (*i.e.*, from 0.77 to 1.13), which is larger in magnitude than the shift from the referent to the first, or first to second groups. Similarly for other PCBs with this pattern—the increase in risk in one region of the exposure-response curve may have large influence on the single slope estimate in the linear exposures model. We explored potential non-linear or non-monotonic relationships between PCB concentration and risk using GAMS. The GAM form tested had 4 degrees of freedom; one degree is taken by the parametric, linear part of the model, and 3 remain for the smoothed spline. In nearly all cases, the linear portion of the association was statistically significant at the alpha = 0.05 level. The spline component was statistically significant for both grouped PCB concentrations (estrogenic, mono-*ortho* substituted) and specific congeners (PCBs 74, 99, 118, 138, 146, 153, 156), indicating non-linearity in the association with risk of hypertension. Most of these showed roughly quadratic relationships, similar to that shown in the partial prediction plot for PCB 138 [Figure 1(a)].

The spline component was not necessarily significant for the PCBs which showed decreased risk in certain exposure categories, but overall increased risk in the linear exposure model, such as PCB 101. As seen in the partial prediction plot for PCB 101 in Figure 1(b), the exposure-response relationship is relatively flat over much of the exposure range, then rises in a relatively linear fashion; thus, although the categorical model results indicated decreased risk in lower exposure ranges, the overall curve does not show a significant departure from linearity. The slope estimate corresponding to the linear portion in the GAM analysis was generally similar (in direction and magnitude) to the slope estimated from the simple linear exposure model (for example, slope estimates of 4.05 in the GAM analysis and 4.05 in the linear exposure model for PCB 52). The exception is PCBs 156 and 157, where the direction and magnitude both changed. The partial prediction plot for PCB 156 (similar in shape to that for PCB 157) in Figure 1(c) shows a relatively flat relationship in the lower exposure range, followed by a steep drop off; for comparison, the partial prediction plot for PCB 138 (similar to the plots for the other PCBs with significant spline components) shows a more gradual change, with increased risk in the low dose region tailing off as exposure increases. The shaded area in each plot is the Bayesian 95% confidence band around the estimate, and indicates greater uncertainty with increasing PCB level; this is expected, since few individuals had very high serum concentrations.

In order to investigate potential multicollinearity among PCB congeners, a logistic regression model was constructed with all PCB congeners included as predictor variables (along with the same potential confounders indentified above). In this model, multiple congeners showed an association with hypertension risk—PCBs 99, 118 and 128 had p-values<0.05, while PCBs 105 and 167 had p-values between 0.05 and 0.10. However, regression diagnostics indicated the presence of multicollinearity between PCBs 157 and 167; PCBs 170 and 180; and PCBs 146 and 153. Therefore, we continued to the next set of analyses to explore potential clustering of variables and data reduction.

Cluster analysis identified 4 clusters of similarly acting PCBs (Table 4). Based on these results, four new variables were created as the sum of the concentrations of the congeners within each cluster. However, although these clusters explained over 80% of the variance, only two of the cluster variables were significant in multivariate logistic regression (p-values of 0.13, 0.001, 0.09 and 0.02). Discriminant analysis identified 12 congeners as the most informative. When including these 12 in a discriminant analysis, 35.33% of cases were classified correctly. Principal component analysis showed that four components had eigenvalues >1; in the first component (eigenvalue of 13.21), there was no single dominant PCB and all weights were positive. Most congeners had weights between 0.2 and 0.3, with a few (PCBs 52, 66, 101, 128) having weights lower than this range. The remaining three components had much smaller eigenvalues (2.14 to 1.03) and a wider range of weights, including both positive and negative values. These four components were entered into a multivariate logistic regression model along with previously identified potential confounders. All of the factors were associated with odds of hypertension, although with borderline statistical significance (p-values ranging from 0.05 to 0.08).

Finally, non-linear optimization was used to construct a maximally informative weighted sum of the standardized PCB concentrations. The initializing parameter values gave equal weight to each congener, and the Newton-Raphson with line search technique was used to determine the weights which maximized the log-likelihood function for the logistic regression model. The congeners with non-zero contributions to the weighted sum were: PCBs 66 (weight = 0.32), 101 (weight = 0.08), 118 (weight = 0.22), 128 (weight = 0.09) and 187 (weight = 0.30). A new variable was constructed to represent this weighted sum of the centered congeners, which was significantly associated with hypertension risk in the multivariate logistic regression model (beta = 0.39 [SE = 0.09], p-value < 0.0001).

As a sensitivity analysis, these procedures were repeated using lipid adjusted PCB serum concentrations rather than including serum lipids as a separate covariate. Results were very similar to those for the main analysis, but in most cases the effect estimates were somewhat attenuated.

### 3.2. Discussion

This study used epidemiologic data demonstrate an approach to analyzing complex exposures. Classical methods in epidemiology may not be adequate, due to the large number of exposures relative to the sample size, correlated exposures, and exposures of varying ranges and potencies. This example begins with standard epidemiologic regression analyses. Further steps are to investigate potential non-linearity of the association between exposure and outcome using splines, and multicollinearity among predictors using regression diagnostics. Next, cluster analysis, discriminant analysis, and principal component approaches are used to identify most informative congeners and clusters of congeners. Finally, non-linear optimization is used to construct a maximally informative weighted sum of the multiple congeners. Each of these analytic approaches has strengths and limitations. Multiple approaches may be used to investigate non-linearity in the exposure-response relationship. We chose to evaluate PCB exposure as a categorical variable and to construct splines to assess non-linearity in relation to hypertension risk. The use of categorical variables is straightforward, but results and interpretation may depend on cut-points selected (which in turn may be dependent upon sample size and distribution of exposure among cases and non-cases). Further, if the study population has no unexposed individuals, the choice of a referent group may be problematic. The use of splines offers an advantage in that it is not necessary to select arbitrary cut-points, but results are less easily interpreted and may depend on knot selection and placement. Evaluating correlation among exposure variables is also important in understanding their cumulative impact on risk of a given outcome. One option is to use regression diagnostics. However, there is no clear definition or cutoffs to identify multicollinearity, and interpretation may depend on model form. As an alternative, discriminant analysis may be used to identify the most informative exposure variables—those which have the greatest ability to discriminate between cases and non-cases. Similarly to regression variable selection, discriminant analysis may use forward, backward or stepwise selection of exposure variables. Results may differ depending on the entry method used and criteria for retention; further, only one variable is entered or removed at a time, which does not account for relationships among variables not already in the model. Two options to identify related exposures are cluster analysis and principal component analysis. Both techniques are commonly used for data reduction—cluster analysis groups variables with the goal of finding clusters that are as correlated as possible within the cluster, and as uncorrelated as possible with variables outside the cluster. However, while this approach identifies correlated variables, it does not necessarily provide insight into which are the most informative among the cluster members, or the best way to combine information from cluster members. As an alternative, principal component analysis creates new, uncorrelated component variables from linear combinations of the original, correlated variables. These new components are structured to explain as much of the variance as possible, through selection of the variables in each component, and the weight assigned to each variable. One limitation of these analyses is sensitivity to scale, for example if variables have different units or ranges of distribution. In addition, the interpretation of the new component variables is not always straightforward. To address this issue of interpretation and provide another alternative, we used a non-linear optimization approach to construct an optimally weighted linear combination of exposure variables. In contrast to principal component analysis, where the goal is to maximize the proportion of variance explained, this approach maximizes the likelihood function associated with the statistical model. The variables are centered prior to analysis, and a linear constraint is imposed that the weights sum to one. This way, the weights assigned provide a sense of ‘how much’ of the exposure-response relationship is due to any one exposure variable. Taken together, these approaches augment traditional epidemiologic analyses in the situation where there are multiple, possibly correlated predictor variables of varying potencies and ranges.

The case study examined PCB body burden and risk of hypertension, since previous studies have provided suggestive evidence for this association. We found that in the NHANES, serum concentrations of PCBs did vary by demographic characteristics and hypertensive status. Serum concentrations tended to be higher among older participants. This is likely due to the restrictions of PCB production in the 1970s, as increasing age reflects a longer period of exposure. There was also variation by body mass, and different patterns by BMI category among men compared to women. This variability may be due to the storage of lipophilic PCBs in adipose tissue; individuals gaining adipose tissue may sequester more PCBs (thus lowering serum levels) while those losing adipose tissue release more PCBs into the bloodstream. Such changes may occur during weight gain or loss, pregnancy and lactation [10–13].

We observed associations between certain PCB congeners and hypertension risk, after controlling for potential confounders. The most informative congeners identified by the weighted sum approach were PCBs 66, 101, 118, 128 and 187, with PCBs 66 and 118 (which have a nearly identical structure) and 187 having the largest weights. Each of these was also statistically significantly associated with risk of hypertension in individual congener-specific models. However, these five congeners do not share a common structure (PCBs 66 and 118 are mono-*ortho* substituted, PCBs 101 and 128 are di-*ortho* substituted, PCB 187 is tri-*ortho* substituted), and two of the five (PCBs 66 and 128) are considered estrogenic. A 2008 study by Everett *et al.* identified seven PCBs (including PCB 118 and 187, although PCB 66 was not examined) associated with hypertension risk in the 1999–2002 NHANES cohort; each of which were also identified as most informative in these analyses. The authors hypothesized that the specific arrangement of chlorine atoms may explain differences in association even among congeners similar in structure or activity [14]. Goncharov *et al.* examined the relationship between total PCBs and blood pressure among Alabama residents living near a PCB production facility, and found significant associations with not only clinical hypertension, but also systolic and diastolic blood pressure [15]. A subsequent study in the same population further examined the relationship between PCB exposure and systolic and diastolic blood pressure, and reported significant associations even among those in the normotensive range [4]. Total PCBs, di-*ortho* and tri- and tetra-*ortho* PCBs were associated with both systolic and diastolic blood pressure, and there were borderline associations between estrogenic PCBs and systolic blood pressure, and mono-*ortho* PCBs and diastolic blood pressure. One consideration in comparing these findings to those of the present study, is the difference in study populations. The NHANES is a population based sample, with generally low levels of exposure to many PCB congeners, while the Alabama population has relatively high exposure to a specific industrial PCB mixture (in addition to background sources of exposure). Although not directly comparable to the present study due to differences in the outcome measured, we also found increased risk of hypertension with increasing levels of each of these groups.

There were some limitations in this analysis. Hypertension status was based on several measures, including physician diagnosis, taking an antihypertensive medication, and measured blood pressure. Some of these measures may be more sensitive and specific than others, so as a sensitivity analysis, we re-defined the hypertensive group to include only those with physician diagnosis with medication, or physician diagnosis without medication but with elevated blood pressure. In this group, increased risk was noted for total PCBs based on categorical analysis (ORs for quartiles 2, 3, and 4 *vs.* quartile 1 of 1.43, 1.59 and 1.40, respectively); this appeared to be due mainly to increased risk associated with the di-ortho substituted PCBs (ORs for quartiles 2, 3, and 4 *vs.* quartile 1 of 1.54, 1.59 and 1.38, respectively). Since the NHANES is a cross-sectional design, it is not possible to establish the temporality between exposure to PCBs and risk of hypertension. PCBs do have a relatively long half-life (on the order of years), so that exposure assessment during the NHANES examination may be representative of exposure during a time period relevant to the etiology of hypertension. When an analyte concentration was below the LOD, the value was replaced with LOD/√2. However, this method has been shown to perform well when the number of non-detects is moderate [16]. The limits of detection have changed with subsequent NHANES cycles (generally becoming lower), which affects the proportion of individuals with non-detectable analyte levels across cycles. We combined three cycles to increase sample size and number of individuals with detectable levels of PCBs, but it is possible that changing detection limits may affect these results. We did not adjust for multiple hypothesis testing (all p-values are uncorrected), since the purpose of this analysis was to present a methodological approach rather than explore the relationship between PCB body burden and risk of hypertension. However, adjustment for multiple testing would decrease the number of significant findings. Finally, the biological mechanisms for observed associations between PCBs and hypertension risk are not clear; this is underscored by the fact that congeners with similar structure or biological activity may not have similar associations with the health outcome, even after attempts to correct for differences in exposure level. Strengths of this analysis include the use of the multiple NHANES cycles to generate robust estimates, consideration of multiple important PCBs, and the ability to control for important covariates.

## 4. Conclusions

Emerging research shows that multiple chemicals may affect the same outcome or group of outcomes, due to structural similarity or related modes of action. We have used the case study of PCB exposure and risk of hypertension to demonstrate one approach to assessing the association between multiple environmental exposures and health outcomes. This approach may be readily applied to other outcomes and sets of exposures, including exposures of different classes (for example, structurally unrelated endocrine disruptors, or common pollutants of a certain industrial process). This flexibility and use of established epidemiologic techniques provide a framework for the evaluation of exposure mixtures in relation to human health.

## Figures and Tables

**Table 1:** Selected demographic characteristics overall and by hypertension status, NHANES 1999–2004.

Characteristic	N (%)		

	Total	Normotensive	Hypertensive
Total	4,119 (100)	2,311 (56.1)	1,808 (43.9)
**Gender**			
Male	1,943 (47.2)	1,066 (46.1)	877 (48.5)
Female	2,176 (52.8)	1,245 (53.9)	931 (51.5)
**Age group**			
20–39 years	1,511 (36.7)	1,274 (55.1)	237 (13.1)
40–49 years	664 (16.1)	442 (19.1)	222 (12.3)
50–59 years	541 (13.1)	268 (11.6)	273 (15.1)
60–69 years	627 (15.2)	185 (8.0)	442 (24.5)
70+ years	776 (18.8)	142 (6.1)	634 (35.1)
**Race/ethnicity**			
NH White	2,124 (51.6)	1,135 (49.1)	989 (54.7)
NH Black	739 (17.9)	358 (15.5)	381 (21.1)
Mexican-American	902 (21.9)	583 (25.2)	319 (17.6)
Other Hispanic	192 (4.7)	119 (5.2)	73 (4.0)
Other/Mixed/Missing	162 (3.9)	116 (5.0)	46 (2.5)
**BMI**			
Underweight	71 (1.8)	50 (2.2)	21 (1.2)
Normal weight	1,250 (31.3)	855 (37.6)	395 (22.9)
Overweight	1,419 (35.5)	805 (35.4)	614 (35.6)
Obese	1,257 (31.5)	563 (24.8)	694 (40.3)

**Table 2:** Distribution of selected PCB congeners* by demographic characteristics, NHANES 1999–2004.

Whole weight concentration (ng/g)	Total	Normotensive	Hypertensive
∑**PCBs**			

Geometric Mean	1.10	0.83	1.61
25th percentile	0.66	0.56	1.01
50th percentile	1.10	0.78	1.68
75th percentile	1.94	1.34	2.60

∑**estrogenic PCBs**			

Geometric Mean	0.13	0.10	0.18
25th percentile	0.09	0.08	0.11
50th percentile	0.12	0.10	0.18
75th percentile	0.21	0.15	0.29

∑**mono-*ortho*sub. PCBs**			

Geometric Mean	0.23	0.18	0.33
25th percentile	0.16	0.15	0.20
50th percentile	0.22	0.18	0.33
75th percentile	0.38	0.25	0.52

∑**di-*ortho*sub. PCBs**			

Geometric Mean	0.75	0.55	1.12
25th percentile	0.42	0.34	0.70
50th percentile	0.75	0.51	1.16
75th percentile	1.38	0.96	1.84

∑**tri/tetra-*ortho*sub. PCBs**			

Geometric Mean	0.11	0.09	0.16
25th percentile	0.08	0.08	0.11
50th percentile	0.11	0.09	0.15
75th percentile	0.18	0.13	0.24

∑**dioxin-like PCBs**			

Geometric Mean	0.38	0.27	0.58
25th percentile	0.20	0.17	0.36
50th percentile	0.39	0.25	0.62
75th percentile	0.72	0.49	0.96

**PCB 138&158**			

% < LOD	17.0	23.2	9.2
Geometric Mean	0.14	0.10	0.22
25th percentile	0.08	0.06	0.12
50th percentile	0.14	0.09	0.23
75th percentile	0.29	0.18	0.40

**PCB 153**			

% < LOD	13.8	19.4	6.6
Geometric Mean	0.20	0.14	0.32
25th percentile	0.10	0.08	0.19
50th percentile	0.21	0.13	0.35
75th percentile	0.41	0.28	0.57

**PCB 180**			

% < LOD	15.1	21.7	6.6
Geometric Mean	0.15	0.10	0.24
25th percentile	0.06	0.05	0.15
50th percentile	0.17	0.09	0.28
75th percentile	0.33	0.23	0.44

* Sum of all non-missing PCBs measured in all 3 cycles: 52, 66, 74, 99, 101, 105, 118, 128, 138/158, 146, 153, 156, 157, 167, 170, 172, 177, 178, 180, 183 and 187. Groupings are as follows. Estrogenic: PCBs 66, 74, 99, 128; Mono-*ortho* substituted: PCBs 66, 74, 105, 118, 156, 157, 167; Di-*ortho* substituted: PCBs 52, 99, 101, 128, 138, 146, 153, 170, 172, 180; Tri- and Tetra-*ortho* substituted: PCBs 177, 178, 183, 187; dioxin-like: PCBs 105, 118, 156, 157, 167, 170, 180.

**Table 3:** Association between serum PCBs and odds of hypertension, NHANES 1999–2004.*

	Categorical exposure OR (95% CI)			Continuous exposure	Continuous exposure, natural log transform	Continuous exposure, centered	GAM–linear component	GAM–spline component
	*Quartile 2 vs. 1*	*Quartile 3 vs. 1*	*Quartile 4 vs. 1*	*β (SE) Wald test p*	*β (SE) Wald test p*	*β (SE) Wald test p*	*β (SE) Wald test p*	*Chi-square p*
**∑PCBs (*all non missing values)***	1.05 (0.83–1.34)	1.09 (0.84–1.43)	**1.38 (1.02–1.87)**	0.0689 (0.0360) 0.0553	**0.1579 (0.0727) 0.0299**	**0.1099 (0.0573) 0.0553**	− −±	− −±
**∑estrogenic PCBs**†	0.92 (0.74–1.16)	1.05 (0.84–1.32)	**1.42 (1.10–1.84)**	**0.7637 (0.3172) 0.0161**	**0.1838 (0.0678) 0.0067**	**0.1375 (0.0573) 0.0164**	**0.8378 (0.2536) 0.0010**	**0.0411**
**∑mono-*ortho*sub. PCBs**†	0.92 (0.73–1.16)	1.13 (0.89–1.42)	**1.60 (1.22–2.10)**	**0.4014 (0.1665) 0.0159**	**0.2044 (0.0686) 0.0029**	**0.1420 (0.0593) 0.0166**	**0.4795 (0.1337) 0.0003**	**0.0141**
**∑di-*ortho*sub. PCBs**†	1.07 (0.83–1.36)	1.06 (0.81–1.40)	1.34 (0.98–1.82)	0.0777 (0.0490) 0.1131	0.1321 (0.0712) 0.0637	0.0877 (0.0553) 0.1131	**0.0974 (0.0407) 0.0169**	0.9438
**∑tri/tetra-*ortho*sub. PCBs**†	1.01 (0.79–1.28)	1.05 (0.82–1.36)	1.29 (0.97–1.71)	**0.7817 (0.3537) 0.0271**	0.0966 (0.0651) 0.1378	**0.1257 (0.0575) 0.0287**	**1.1294 (0.3447) 0.0011**	0.6920
**∑dioxin-like PCBs**†	1.01 (0.79–1.29)	1.05 (0.80–1.39)	1.26 (0.91–1.74)	0.1725 (0.0986) 0.0803	**0.1439 (0.0710) 0.0429**	0.0922 (0.0574) 0.0837	− −±	− −±
**Individual congeners**								
	*Tertile 1 vs. <LOD*	*Tertile 2 vs. <LOD*	*Tertile 3 vs. <LOD*	*β (SE) Wald test p*	*β (SE) Wald test p*	*β (SE) Wald test p*	*β (SE) Wald test p*	*Chi-square p*
**PCB 52**	0.85 (0.66–1.08)	**0.77 (0.60–0.98)**	0.87 (0.68–1.12)	**4.2542 (2.0166) 0.0349**	**0.1083 (0.0674) 0.1081**	**0.0996 (0.0469) 0.0337**	**4.0520 (2.0305) 0.0461**	0.7483
**PCB 66**	0.95 (0.74–1.21)	**0.77 (0.60–0.97)**	1.13 (0.88–1.45)	**8.6799 (2.4294) 0.0004**	**0.1539 (0.0566) 0.0065**	**0.2330 (0.0650) 0.0003**	**8.8179 (2.4416) 0.0003**	0.4479
**PCB 74**	1.03 (0.81–1.29)	1.20 (0.94–1.54)	**1.61 (1.21–2.15)**	**1.5528 (0.6253) 0.0130**	**0.2002 (0.0631) 0.0015**	**0.1402 (0.0578) 0.0154**	**2.0481 (0.5724) 0.0004**	**0.0232**
	*Quartile 2 vs. 1*	*Quartile 3 vs. 1*	*Quartile 4 vs. 1*	*β (SE) Wald test p*	*β (SE) Wald test p*	*β (SE) Wald test p*	*β (SE) Wald test p*	*Chi-square p*
**PCB 99**	0.88 (0.71–1.10)	0.90 (0.72–1.12)	1.05 (0.82–1.35)	0.6639 (0.6115) 0.2776	0.0799 (0.0593) 0.1781	0.0516 (0.0490) 0.2924	**1.4130 (0.6007) 0.0187**	**0.0111**
**PCB 101**	**0.77 (0.59–0.99)**	0.90 (0.70–1.16)	**0.71 (0.55–0.92)**	**4.3306 (1.8184) 0.0172**	0.0588 (0.0492) 0.2323	**0.1059 (0.0444) 0.0172**	3.6032 (2.0645) 0.0810	0.3580
**PCB 105**	0.87 (0.69–1.11)	0.90 (0.72–1.13)	**1.25** (0.97–1.62)	**4.4674 (1.7956) 0.0128**	**0.1488 (0.0530) 0.0050**	**0.1634 (0.0664) 0.0139**	**4.4639 (1.4354) 0.0019**	0.1009
**PCB 118**	1.00 (0.79–1.27)	1.12 (0.86–1.44)	**1.63 (1.23–2.17)**	**1.1521 (0.3925) 0.0033**	**0.2206 (0.0547) <0.0001**	**0.1841 (0.0643) 0.0042**	**1.2740 (0.3047) <0.0001**	**0.0175**
**PCB 128**	0.79 (0.50–1.25)	0.82 (0.52–1.27)	0.87 (0.55–1.37)	**10.5785 (3.4915) 0.0024**	**0.0631 (0.0237) 0.0077**	**0.1190 (0.0393) 0.0025**	**11.0304 (3.5228) 0.0018**	0.3670
**PCB 138&158**	1.11 (0.86–1.44)	1.08 (0.83–1.41)	1.21 (0.90–1.61)	0.2080 (0.1833) 0.2567	0.0922 (0.0597) 0.1224	0.0609 (0.0518) 0.2398	**0.3630 (0.1587) 0.0223**	**0.0046**
**PCB 146**	1.01 (0.81–1.26)	1.06 (0.84–1.32)	**1.48 (1.15–1.91)**	**2.3435 (1.0986) 0.0329**	0.0903 (0.0611) 0.1393	**0.1219 (0.0566) 0.0312**	**3.4321 (1.0103) 0.0007**	**0.0257**
**PCB 153**	1.05 (0.79– 1.39)	1.26 (0.94–1.68)	**1.42 (1.03–1.95)**	0.2321 (0.1457) 0.1112	**0.1199 (0.0609) 0.0489**	0.0903 (0.0552) 0.1021	**0.3631 (0.1221) 0.0030**	**0.0183**
**PCB 156**	1.00 (0.80–1.25)	1.00 (0.79–1.28)	1.24 (0.94–1.62)	−0.2324 (0.6936) 0.7376	0.0282 (0.0587) 0.6308	−0.0183 (0.0421) 0.6627	**2.2396 (0.9765) 0.0219**	**0.0082**
**PCB 157**	1.01 (0.78–1.32)	**0.74 (0.57–0.95)**	0.81 (0.61–1.07)	−1.7276 (2.7168) 0.5249	0.0136 (0.0430) 0.7512	−0.0264 (0.0382) 0.4895	**3.9313 (3.6762) 0.2850**	0.0610
**PCB 167**	0.91 (0.70–1.19)	**0.71 (0.55–0.92)**	1.31 (0.96–1.78)	2.8837 (2.9219) 0.3237	0.0449 (0.0380) 0.2377	0.0453 (0.0470) 0.3350	3.9883 (2.6270) 0.1290	0.0593
	*Quartile 2 vs. 1*	*Quartile 3 vs. 1*	*Quartile 4 vs. 1*	*β (SE) Wald test p*	*β (SE) Wald test p*	*β (SE) Wald test p*	*β (SE) Wald test p*	*Chi-square p*
**PCB 170**	1.07 (0.83–1.38)	1.14 (0.86–1.51)	1.36 (0.99–1.86)	0.8607 (0.5392) 0.1104	0.1117 (0.0662) 0.0913	0.0729 (0.0547) 0.1826	**1.2980 (0.4727) 0.0061**	0.0764
**PCB 172**	0.90 (0.70–1.15)	0.83 (0.65–1.05)	1.17 (0.90–1.54)	**6.2173 (2.9162) 0.0330**	0.0433 (0.0456) 0.3425	**0.1052 (0.0506) 0.0378**	**8.0991 (2.9156) 0.0055**	0.6976
**PCB 177**	0.93 (0.72–1.19)	0.85 (0.67–1.06)	0.95 (0.73–1.24)	2.0869 (2.2230) 0.3479	0.0126 (0.0548) 0.8184	0.0428 (0.0487) 0.3794	3.2771 (2.0929) 0.1175	0.0700
**PCB 178**	0.92 (0.72–1.17)	0.87 (0.69–1.10)	1.21 (0.93–1.58)	4.6817 (2.5458) 0.0659	0.0401 (0.0520) 0.4405	0.0925 (0.0508) 0.0683	**6.1459 (2.7717) 0.0267**	0.2531
**PCB 180**	0.95 (0.72–1.25)	1.06 (0.79–1.44)	1.22 (0.86–1.72)	0.2610 (0.2019) 0.1960	0.0864 (0.0617) 0.1617	0.0664 (0.0542) 0.2210	**0.4762 (0.1874) 0.0111**	0.3838
**PCB 183**	1.01 (0.81–1.26)	0.95 (0.76–1.18)	1.02 (0.80–1.31)	3.5053 (1.9062) 0.0659	0.0439 (0.0578) 0.4470	0.0933 (0.0520) 0.0729	**4.8404 (1.9036) 0.0110**	0.2414
**PCB 187**	1.12 (0.89–1.42)	1.16 (0.89–1.50)	1.25 (0.93–1.67)	**1.4990 (0.5978) 0.0122**	0.1178 (0.0603) 0.0510	**0.1517 (0.0598) 0.0111**	**2.1694 (0.5855) 0.0002**	0.5394

* Categorical variable is based on quartiles (quartile 1 is the referent) for summed congeners, and below the LOD (referent), and tertiles among those above the LOD for individual congeners. All models include the following covariates: age in years, sex, BMI at exam, race/ethnicity, current smoking status, regular physical activity status, family history of cardiovascular disease, total cholesterol and serum lipid concentration.

† Groupings are as follows. Estrogenic: PCBs 66, 74, 99, 128; Mono-*ortho* substituted: PCBs 66, 74, 105, 118, 156, 157, 167; Di-*ortho* substituted: PCBs 52, 99, 101, 128, 138, 146, 153, 170, 172, 180; Tri- and Tetra-*ortho* substituted: PCBs 177, 178, 183, 187; dioxin-like: PCBs 105, 118, 156, 157, 167, 170, 180. ± GAM model did not converge due to numerical instability; no results available.

**Table 4:** Results from collinearity and grouping analyses.

Analytic approach	Results
Collinearity	Collinearity present between: PCBs 157 and 167; PCBs 170 and 180; PCBs 146 and 153
Cluster analysis	4 clusters identified: PCBs 138, 146, 153, 170, 172, 177, 178, 180, 183, and 187; PCBs 52, 66, 101 and 128; PCBs 74, 99, 105 and 118; PCBs 156, 157 and 167
Discriminant analysis	Most strongly associated PCBs are: 66, 74, 99, 105, 118, 128, 156, 157, 167, 178, 180 and 187
Principal component analysis	4 components with eigenvalues >1.0
Optimization of weighted sum	PCBs 66 (weight = 0.3163), 101 (weight = 0.0819), 118 (weight = 0.2183), 128 (weight = 0.0856) and 187 (weight = 0.2979)
